# Quality of life and adherence to treatment in early-treated Brazilian phenylketonuria pediatric patients

**DOI:** 10.1590/1414-431X20176709

**Published:** 2017-12-11

**Authors:** E. Vieira, H.S. Maia, C.B. Monteiro, L.M. Carvalho, T. Tonon, A.P. Vanz, I.V.D. Schwartz, M.G. Ribeiro

**Affiliations:** 1 Agência Nacional de Saúde Suplementar, Rio de Janeiro, RJ, Brasil; 2 Instituto de Puericultura e Pediatria Martagão Gesteira, Universidade Federal do Rio de Janeiro, Rio de Janeiro, RJ, Brasil; 3 Departamento Materno-Infantil, Faculdade de Medicina, Universidade Federal Fluminense, Niterói, RJ, Brasil; 4 Núcleo de Estudos da Saúde do Adolescente, Universidade do Estado do Rio de Janeiro, Rio de Janeiro, RJ, Brasil; 5 Instituto Estadual de Diabetes e Endocrinologia Luiz Capriglione, Rio de Janeiro, RJ, Brasil; 6 Serviço de Genética Médica, Hospital de Clínicas de Porto Alegre, Porto Alegre, RS, Brasil; 7 Programa de Pós Graduação em Ciências Médicas, Universidade Federal do Rio Grande do Sul, Porto Alegre, RS, Brasil; 8 Departamento de Genética, Universidade Federal do Rio Grande do Sul, Porto Alegre, RS, Brasil

**Keywords:** Phenylketonuria, Quality of life, Questionnaires, Patient compliance, Diet therapy

## Abstract

Early dietary treatment of phenylketonuria (PKU), an inborn error of phenylalanine (Phe) metabolism, results in normal cognitive development. Although health-related quality of life (HRQoL) of PKU patients has been reported as unaffected in high-income countries, there are scarce data concerning HRQoL and adherence to treatment of PKU children and adolescents from Brazil. The present study compared HRQoL scores in core dimensions of Brazilian early-treated PKU pediatric patients with those of a reference population, and explored possible relationships between adherence to treatment and HRQoL. Early-treated PKU pediatric patient HRQoL was evaluated by self- and parent-proxy reports of the Pediatric Quality of Life Inventory (PedsQL) core scales. Adherence to treatment was evaluated by median Phe levels and percentage of results within the therapeutic target range in two periods. Means for total and core scales scores of PedsQL self- and parent proxy-reports of PKU patients were significantly lower than their respective means for controls. Adequacy of median Phe concentrations and the mean percentage of values in the target range fell substantially from the first year of life to the last year of this study. There was no significant difference in mean total and core scale scores for self- and parent proxy-reports between patients with adequate and those with inadequate median Phe concentrations. The harmful consequences for intellectual capacity caused by poor adherence to dietary treatment could explain the observed decrease in all HRQoL scales, especially in school functioning. Healthcare system and financial difficulties may also have influenced negatively all HRQoL dimensions.

## Introduction

Phenylketonuria (PKU, OMIM 261600) is an autosomal recessive inborn error of phenylalanine metabolism, caused by a deficient activity of phenylalanine hydroxylase (PAH, EC 1.14.16.1) – an enzyme that coverts the essential amino acid phenylalanine (Phe) to tyrosine, leading to an accumulation of Phe in blood and other tissues (1). When untreated, most individuals with PKU develop severe intellectual disability, microcephaly, neurological disorders, eczema and decreased hair and skin pigmentation (2). In Latin America, it was estimated an incidence of PKU of about 1:21,000 live births (1:12,000 to 1:52,000) (3). In Brazil, the incidence of PKU was reported as approximately 1 in 25,000 live births (4), although there are important variations among different Brazilian states, from 1:9,000 to 1:33,000 (5–9).

The establishment of mass newborn screening programs allowed the detection of PKU in the neonatal period and the early establishment of dietary treatment, resulting in a normal cognitive development (10). In 2001, the National Neonatal Screening Program was established within the Brazilian National Health System, ensuring access of all newborns to screening, confirmatory tests, and treatment for PKU, including the provision of metabolic phenylalanine-free formula (4).

The need to adhere to a strict diet, frequent blood sampling to monitor the levels of Phe and regular visits to health services, can affect daily life and therefore have a negative impact on the health-related quality of life (HRQoL) of individuals affected by PKU (11–13). In addition, there is evidence that adherence to diet treatment in early-treated PKU patients deteriorates with increasing age, being unsatisfactory in older children and adolescents (14). Low adherence to treatment, besides its negative consequences for the psychomotor development of pediatric patients, could also reduce their HRQoL, as has been suggested for adults (15,16).

The present study intends to contribute to the knowledge of HRQoL of early-treated pediatric patients in Brazil, investigating differences in core dimensions of this outcome measure between them and a reference healthy schoolchildren population, and exploring possible relationships between adherence to treatment and HRQoL.

## Material and Methods

### Study design

The study was conducted from March 2012 to July 2014. Parents of patients with early-treated PKU, ranging in age from 6 to 18 years, followed in two centers in Brazil – State Institute of Diabetes and Endocrinology Luiz Capriglione (IEDE), Rio de Janeiro, RJ, and Medical Genetics Service, Hospital de Clínicas de Porto Alegre (HCPA), Porto Alegre, RS, were invited to participate in the study. The affected individuals should have had a diagnosis of classic, moderate or mild PKU in the first four months of life, and since then been submitted to a protein-restricted diet supplemented with a Phe-free amino acid formula. Classic PKU was defined as the finding of pre-treatment blood Phe levels repeatedly >1200 μmol/L (>20 mg/dL), whereas moderate and mild PKU were characterized by pre-treatment blood Phe levels in the ranges of 900–1200 μmol/L (15–20 mg/dL), and 600–900 μmol/L (10–15 mg/dL), respectively (17). They might have presented varying degrees of treatment adherence and metabolic control, but all participants had their blood Phe levels regularly monitored.

The following exclusion criteria were adopted: 1) inability of children and parents in understanding the study questionnaires; 2) presence of severe chronic or disabling diseases unrelated to PKU.

### Participants' demographics

A total of 105 affected pediatric patients fulfilled the eligibility criteria, of which 63 were followed at IEDE and 42 at HCPA. Fifty-one of the 105 eligible children and adolescents with PKU were recruited and accepted to participate in the study (response rate 49%), including 38 followed at IEDE and 13 at HCPA. The sample included 33 classic PKU and 17 mild or moderate PKU patients (for 1 patient this information could not be obtained). The mean age at diagnosis was 45±22 days, and the mean age at the start of treatment with a Phe-restricted diet was 48±20 days.

Thirteen of 38 children and adolescents from Rio de Janeiro evaluated by RCPM and RSPM had their intellectual capacity classified as Grade IV (definitely below average in intellectual capacity) or Grade V (intellectually defective). Intellectual capacity of 1 of 13 patients from Rio Grande do Sul was evaluated clinically as defective with no formal psychometric test being applied.

Twenty-nine patients lived in Metropolitan areas, while 22 lived in the countryside of both states. Twenty-two females and 29 males were enrolled in the study. Twenty-five children and adolescents belonged to middle-income families (class C), 20 to high-income families (classes A and B), and 2 to low-income families (class D) – four families did not fill the socioeconomic status evaluation form.

The gender distribution of our sample of patients did not differ from that of the normative sample of healthy school children and adolescents (21) (χ^2^=1.2240, P=0.269). The socioeconomic status distribution of our sample of patients differed from the normative sample (χ^2^=6.7518, P=0.034), due to the higher percentage of high-income families in our sample – 42.6 *vs* 31.1%. Nevertheless, the socioeconomic status distribution of our PKU patient sample did not differ significantly from those of the populations of Rio de Janeiro (χ^2^=7.6612, P=0.176) and Porto Alegre (χ^2^=2.5856, P=0.764) metropolitan areas (22).

### Ethical issues

This study was approved by the Research Ethics Review Committee of IEDE and HCPA. At all times, the study was conducted under ethical principles and in line with the Guidelines and Standards for Research in Human Beings, established by Resolution No. 466/2012 of the Brazilian National Health Council (18). Informed consent was obtained from the parents or guardians of all pediatric patients engaged in this study. Active consent was then obtained from each individual child and adolescent whose parents agreed to his/her participation.

### Quality of life and socioeconomic status questionnaires, and intellectual capacity tests

Pediatric Quality of Life Inventory (PedsQL) Version 4.0 is a generic questionnaire for assessing HRQoL of children and adolescents aged 2 to 18 years, originally developed by Varni et al. (19). Its core scales are designed to evaluate four dimensions – physical health, emotional functioning, social functioning, and school functioning. The aggregation of emotional, social and school dimensions is the psychosocial health summary score (19). Items of the questionnaire are Likert scales that are reverse-scored and linearly transformed to a 0–100 scale, where higher scores represent better quality of life. Self-assessment and parent proxy forms of the questionnaire were employed according to the child or adolescent's age.

Normative data from healthy children and adolescents were collected in the city of São Paulo, SP, Brazil, by the authors of the translation and validation to Brazilian Portuguese of the original English version of PedsQL 4.0, which results have been published elsewhere (20,21). This reference population included 240 healthy children and adolescents from 2 to 18 years of age living in urban areas of the outskirts of Greater São Paulo, and their respective parents. Enrolment locations were public schools for children aged 5 to 18 and primary healthcare units for children aged 2 to 4. Inclusion criteria included ability to answer the HRQoL questionnaire, absence of chronic or acute illness in the last 30 days before the interview, and presence of at least 1 parent on the day the questionnaire was applied.

Socioeconomic status evaluation of the families of children and adolescents with PKU was carried out according to the criteria of the Brazilian Association of Research Companies (22). For comparison purposes, these criteria were simplified to three classes: low-income (classes D and E), middle-income (classes C1 and C2), and high-income classes (classes A1, A2, B1, and B2).

Intellectual capacity of children aged 6 to 11 years was evaluated by Raven's Colored Progressive Matrices - RCPM (23), and of adolescents aged 12 to 18 years by Raven's Standard Progressive Matrices - RSPM (24). These are non-verbal intelligence tests developed by John C. Raven in 1936 in the United Kingdom, which employ abstract geometrical figures to directly measure general intelligence, and avoid influences of cultural elements and training. In the present study, we used the Brazilian standardizations of RCPM and of RSPM. The Brazilian standardization of RCPM is based on the results of 1547 kindergarten and elementary school children, aged 5 to 11.5 years, from the city of São Paulo (25), and of RSPM is based on 366 individuals with educational level ranging from elementary to postgraduate school, from the city of Rio de Janeiro (24).

### Adherence to treatment

Adherence to treatment was measured indirectly by the median Phe level in the first year of life and in the last year of treatment evaluated by this study (2010–012). We also evaluated, for both periods, the percentage of blood Phe results in the treatment target range according to the Brazilian PKU Guidelines (26). Adherent children and adolescents were defined as those with median Phe levels ≤6 mg/dL in the first year of life, or ≤10 mg/dL for those ≥13 years of age and ≤6 mg/dL for those <13, in the last year of the study.

### Statistical analyses

The *t*-test for two independent samples was conducted between the normative pediatric data and the results of the self-assessment and parent proxy reports for the PedsQL core scales of PKU children and adolescents in this study. This same test was employed to evaluate potential significant differences in HRQoL between states or area of residence (Rio de Janeiro and Porto Alegre Metropolitan areas or countryside), gender, and severity of disease (classic or mild PKU). One-way ANOVA was used to assess differences in HRQoL scores due to socioeconomic status. A multi-way ANOVA taking into account state or area of residence, gender, disease severity, and socioeconomic status was also performed.

Associations between scales of the PedsQL dimensions for self-assessment and parent proxy reports and the adherence to treatment variables were examined by linear regression analysis. Two independent samples *t*-test was conducted between the results of the self-assessment and parent proxy reports of the PedsQL scales for adherent and non-adherent PKU children and adolescents. A multi-way ANOVA taking into account adherence to treatment in the first year of life and in the last year of the study and the other previously mentioned independent variables was also carried out.

All statistical analyses were conducted with Stata/SE 12.1 for Mac software package (StataCorp, USA).

### Minimum sample size

The minimum sample size was calculated from the previously published mean scores and standard deviations for the PedsQL core scales results of the self-assessment and parent proxy reports of Brazilian healthy children and children with rheumatic diseases (20). A minimum sample size of 35 children in each group was considered in order to detect a difference of ten points on a hypothetical scale, the variance of which was the mean of the weighted variances of the physical, emotional, social and school scales of the self-assessment and parent proxy reports (219.5), accepting an alpha error of 5% and a beta error of 20%.

## Results

### HRQoL

PedsQL self-reports were administered to 49 early-treated PKU patients aged 6 to 17 years – self-reports could not be administered to 2 patients (1 refused to answer the questionnaire and the other was not able to fill it out owing to intellectual disability). Means for total score and for the scores from physical health, emotional functioning, social functioning, school functioning, and psychosocial health scales for PedsQL self-reports were significantly lower than the respective means from the normative sample of healthy schoolchildren (Table 1).

**Table 1.** Self-reported scores for PedsQL 4.0 generic core scales of Brazilian early-treated phenylketonuria (PKU) pediatric patients.

Aspect	Items	PKU (n=49)	Controls (n=180)	P
Total score	23	75.31±12.04	88.90±7.35	<0.0001
Physical health	8	82.54±14.67	95.94±5.83	<0.0001
Emotional functioning	5	66.84±18.31	73.03±16.52	0.0241
Social functioning	5	79.29±22.52	93.14±10.54	0.0001
School functioning	5	68.27±17.00	89.31±11.80^a^	<0.0001
Psychosocial health	15	71.44±13.73	85.03±9.66	<0.0001

Data are reported as means±SD. The *t*-test for two independent samples considering as controls a group of healthy school children of the city of São Paulo, Brazil was used.

a n=173.

In addition, PedsQL parent proxy-reports were administered to 34 parents and caretakers of early-treated PKU children and adolescents. Most of these parents and caretakers (33) were from Rio de Janeiro, which corresponds to 86.8% of this state sample. With the exception of emotional functioning scale, mean scores for the generic core scales for parent proxy-reports were significantly lower than the respective means for parent proxy-reports from the normative sample (Table 2).

**Table 2.** Parent-proxy scores for PedsQL 4.0 generic core scales of Brazilian early-treated phenylketonuria (PKU) pediatric patients.

Aspect	Items	PKU (n=34)	Controls n=240)	P
Total score	23	79.98±16.26	92.32±6.01	0.0001
Physical health	8	89.62±16.71	97.86±4.31	0.0072
Emotional functioning	5	73.97±20.59	80.52±12.59	0.0789^a^
Social functioning	5	84.85±21.93	96.38±8.89	0.0046
School functioning	5	65.74±24.25	90.93±11.85^b^	<0.0001
Psychosocial health	15	74.85±18.24	89.18±8.19	0.0001

Data are reported as means±SD. The *t*-test for two independent samples considering as controls a group of healthy school children of the city of São Paulo, Brazil was used.

a Non-significant difference.

b n=207.

The mean total score for PedsQL self-reports of children and adolescents from Rio de Janeiro (n=37) was not significantly different from the mean of their peers from Rio Grande do Sul (n=12, t=0.5911, P=0.5573). We did not find significant differences between pediatric patients from the two states with regard to emotional functioning (t=0.4868, P=0.6287), social functioning (t=−0.7870, P=0.4352), school functioning (t=−0.6960, P=0.4899), and psychosocial health (t=−0.4910, P=0.6257) scores. Unexpectedly, the patients from Rio de Janeiro evaluated their physical health more positively than their peers from Rio Grande do Sul (t=2.3615, P=0.0224). This difference was upheld even when other independent variables such as region, gender, socioeconomic class, and disease severity were taken into account in a multi-way ANOVA model (F=11.85, P=0.0014). The number of parent proxy-reports from Rio Grande do Sul (n=1) was too small to allow any statistical inference.

In addition, children and adolescents from the interior of both states (n=22) evaluated their global quality of life similar to those living in the metropolitan areas (n=29) (t=−0.8086, P=0.4228). Mean scores for all the generic self-report core scales did not differ significantly between countryside and metropolitan area pediatric patients.

Parent proxy-reports from Rio de Janeiro pediatric patients also did not show any significant difference between metropolitan area (n=23) and countryside (n=10) concerning PedsQL total score (t=−0.6348, P=0.5302). Mean scores for all the generic parent proxy-report core scales did not differ significantly between patients from countryside and metropolitan areas of Rio de Janeiro.

We did not find any significant gender difference concerning the self-perception (t=−1.3172, P=0.1942) and the parents' perception (t=−0.8625, P=0.3948) of quality of life assessed by PedsQL self-report and parent proxy-report total scores. But girls self-evaluated their emotional functioning more negatively than boys (t=−2.8485, P=0.0065). This was the only PedsQL core scale that showed any gender difference for self-reports, but this difference tended to disappear when other independent variables were included in a multi-way ANOVA model (F=3.06, P=0.0884). Considering exclusively parent proxy-reports, there was no gender difference in any PedsQL core scale.

Mean total score for PedsQL self-reports of children and adolescents with mild or moderate PKU was not significantly different compared to pediatric patients affected by classic PKU (t=−0.1901, P=0.8501). Physical health (t=−0.2400, P=0.8114), emotional functioning (t=0.6868, P=0.4957), social functioning (t=−0.5163, P=0.6081), school functioning (t=−0.3594, P=0.7210), and psychosocial health (t=−0.1240, P=0.9019) self-report scores did not show any significant difference between mild or moderate and classic PKU pediatric patients either.

Nevertheless, the mean total score for PedsQL parent-reports of children and adolescents with mild or moderate PKU was significantly higher than that of parent-reports of pediatric patients affected by classic PKU (t=−2.5012, P=0.0189). This difference tended to disappear when other independent variables (region, socioeconomic class, and disease severity) were included in a multi-way ANOVA model (F=3.97, P=0.0561). Similar differences were also found concerning physical health (t=−2.2383, P=0.0323), and school functioning (t=−2.0841, P=0.0452) in parent-report scores. However, only mean score for school functioning in parent-reports was persistently lower in classic PKU (62.2) than mild or moderate PKU (80.6) in a multi-way ANOVA model (F=4.42, P=0.0446). No significant differences were found between the two groups with regard to emotional functioning (t=−1.2524, P=0.2195), social functioning (t=−1.0938, P=0.2838), and psychosocial health (t=−1.6988, P=0.0991) scores.

The number of low-income class families of PKU patients (classes D and E, n=2) was too small for making any significant conclusions. Thus, the assertions regarding socioeconomic classes refer to differences between middle-income – C (n=24) and high-income – A and B (n=20) classes. There was no significant difference in mean total score for PedsQL self-reports of children and adolescents among different socioeconomic classes (F=0.68, P=0.5113). Physical health (F=0.20, P=0.8199), social functioning (F=0.18, P=0.8367), school functioning (F=0.93, P=0.4014), and psychosocial health (F=0.79, P=0.4596) self-report scores also did not show any significant difference among socioeconomic classes, but surprisingly children and adolescents from middle-income class evaluated their emotional functioning more positively than their high-income class peers (F=6.60, P=0.0032). This difference was retained even when other independent variables were considered in a multi-way ANOVA model (F=11.29, P=0.0018).

With reference to the parent proxy-reports, we also did not find a significant difference in mean total score among different socioeconomic classes (t=0.5448, P=0.5898). Physical health (t=0.6749, P=0.5070), emotional functioning (t=−0.0050, P=0.9960), social functioning (t=0.3839, P=0.7037), school functioning (t=0.6228, P=0.5379), and psychosocial health (t=0.4210, P=0.6767) parent proxy-report scores also did not show any significant difference among socioeconomic classes.

### Adherence to treatment

**Figure 1.**
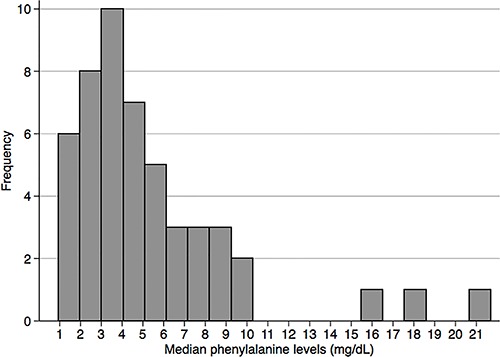
Median phenylalanine levels in the first year of life of Brazilian early-treated PKU pediatric patients. Thirty percent of the patients had median phenylalanine levels in the first year of life above the upper limit of the recommended target range for this age (6 mg/dL). PKU: phenylketonuria.

The mean value for the median Phe concentration in the first year of life was 5.3±4.1 mg/dL. Thirty-five children (70.0%) had adequate median Phe concentrations at this age, i.e., below the upper limit of the target range for children 0 to 12 months of age – 2 to 6 mg/dL, according to the Brazilian PKU Guidelines (26). The distribution of the median Phe concentrations in the first year of life is depicted in Figure 1. The mean percentage of values in the target range was 61.3±28.9% in this period. There was no significant difference in median Phe concentrations in the first year of life among pediatric patients of the two states (t=−0.6663, P=0.5087) or of the two regions – countryside and metropolitan areas (t=1.4452, P=0.1549) included in the present study. Gender (t=0.0888, P=0.9296) and severity of disease (t=1.0046, P=0.3203) also did not influence this adherence parameter. The median Phe concentrations in the first year of life of middle-income class patients did not differ significantly from those of high-income class patients (t=−0.3114, P=0.7571). Similar results were found for the other parameter of adherence in the first year of life (percentage of values in the target range).

In the last year of treatment evaluated by this study (2010–2012) the mean value for the median Phe concentration increased to 9.2±4.3 mg/dL, and the percentage of children with adequate median Phe concentrations fell to 31.4% (16/51). The mean percentage of values in the target range also dropped to 30.9±36.7% in the last year of treatment. There was no significant difference in median Phe concentrations in the last year of treatment among pediatric patients of the two states (t=0.4737, P=0.6378) or of the countryside and metropolitan areas (t=−0.8735, P=0.3867) included in the present study. Gender (t=0.4941, P=0.6235), disease severity (t=1.9745, P=0.0541), and socioeconomic status (t=0.4676, P=0.6424) also did not influence this adherence parameter. Similar results were found for percentage of values in the target range.

No statistically significant association was found between scores for self-report PedsQL generic core scales and the four ‘adherence to treatment’ variables by linear regression analysis (Table 3).

**Table 3.** Linear regression analysis of the self-report scores for PedsQL 4.0 generic core scales and adherence to treatment variables of Brazilian early-treated phenylketonuria (PKU) pediatric patients.

Aspect (dependent variable)	Independent adherence to treatment variables							
	Median Phe 1st year		% Phe results in the target range 1st year		Median Phe last year		% Phe results in the target range last year	
	*t* a	P	*t*	P	*t*	P	*t*	P
Total score	−0.93	0.356	−0.14	0.891	−0.70	0.488	0.33	0.747
Physical health	−1.28	0.208	1.67	0.102	−0.45	0.653	0.30	0.766
Emotional functioning	0.34	0.733	−1.45	0.154	0.21	0.831	−0.21	0.832
Social functioning	−0.41	0.685	−0.60	0.550	−0.91	0.365	0.29	0.776
School functioning	−1.19	0.242	−0.42	0.678	−0.66	0.512	0.49	0.630
Psychosocial health	−0.53	0.599	−1.13	0.264	−0.68	0.501	0.27	0.791

Phe: phenylalanine.

a *t*-statistic.

**Figure 2.**
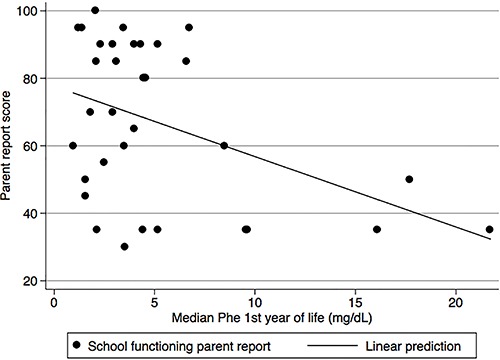
Parent proxy-report school functioning score and median phenylalanine (Phe) levels in the first year of life. A significant inverse relationship between these two variables was found by linear regression analysis (t=−2.64, P=0.013).

There was a significant inverse relationship between parent proxy-report school functioning score and median Phe levels in the first year of life (t=−2.64, P=0.013; Figure 2). No other statistically significant association was found between scores for parent proxy-report PedsQL generic core scales and the four ‘adherence to treatment’ variables by linear regression analysis (Table 4)

**Table 4.** Linear regression analysis of the parent proxy-report scores for PedsQL 4.0 generic core scales and adherence to treatment variables of Brazilian early-treated phenylketonuria (PKU) pediatric patients.

Aspect (dependent variable)	Independent adherence to treatment variables							
	Median Phe 1st year		% Phe results in the target range 1st year		Median Phe last year		% Phe results in the target range last year	
	*t* a	P	*t*	P	*t*	P	*t*	P
Total score	−1.31	0.200	0.49	0.630	−0.42	0.679	−0.48	0.632
Physical health	−0.83	0.415	0.50	0.617	−0.53	0.598	−0.40	0.689
Emotional functioning	−0.71	0.484	−0.53	0.603	0.20	0.844	−0.51	0.616
Social functioning	−0.17	0.869	−0.15	0.878	0.56	0.579	−1.55	0.130
School functioning	−2.64	0.013*	1.64	0.111	−1.41	0.169	0.74	0.465
Psychosocial health	−1.39	0.175	0.42	0.676	−0.31	0.760	−0.47	0.643

Phe: phenylalanine.

a *t*-statistic.

* P≤0.05.

Means for all self-report and parent proxy-report scores of PedsQL generic core scales of adherent PKU children and adolescents were not different from the respective means of their non-adherent peers, both in the first year of life and in the last year of the study (Supplementary Table S1). A multi-way ANOVA taking into account adherence to treatment in these two periods and the other previously mentioned independent variables did not show any significant difference attributable to adherence for the self-report and parent proxy-report PedsQL generic core scales score of adherent and non-adherent PKU pediatric patients (Supplementary Table S2).

## Discussion

A generic instrument of pediatric HRQoL, PedsQL, was chosen to ascertain the impact of PKU and its treatment on the daily life of affected individuals, in their physical, emotional, social, and school dimensions, allowing the cross-comparison to a normal healthy population of school-age children. Generic instruments of HRQoL permit comparisons between different populations (27), and consequently are indispensable for exploratory studies as ours. Disease-specific instruments of HRQoL are generally more sensitive than generic versions for assessing the impact of particular interventions, and are essential to demonstrate small changes over time in HRQoL, for example after the start of BH4 treatment (27,28). Nonetheless, Brazilian and European Portuguese versions of the PKU-specific HRQoL questionnaire, PKUQOL (28,29), were not available during the execution of this study.

Lower scores were obtained for almost all the generic core scales for PedsQL self-reports and parent-proxy reports of PKU children and adolescents when compared to a normative sample of healthy Brazilian schoolchildren. This definitely is in contrast to the findings of most studies done in high-income countries that demonstrated that HRQoL of patients with PKU is similar to that of the general population (11,12,28,30,31). PedsQL was employed only in one of these studies (28), a recent multicenter European study that showed that HRQoL scores of children, adolescents, and adults with PKU evaluated by generic instruments were in the same range as those established as reference values for the US population.

This striking difference between our findings and those of the studies from high-income countries could be attributed, in the case of the European multicenter study (28), to the access to pharmacological treatment with BH4, as increased Phe tolerance and reduced metabolic formula requirement in BH4-responsive patients can improve HRQoL (32). Nevertheless, this cannot be sustained as the other four studies were conducted before BH4 became available. Difficulties faced by the Brazilian National Health System in ensuring a regular supply of PKU metabolic formula to patients, and the financial burden of out-of-pocket acquisition of low protein food by families may have a negative impact on HRQoL of Brazilian PKU patients (33).

The relatively low response rate of the present study - 49%, compared to the studies of PKU patients in European countries, ranging from 59 to 91% (11,12,28,30,31), could not explain the lower HRQoL of Brazilian PKU children and adolescents, since the included patients were those that regularly attended the scheduled metabolic and nutritional consultations, and therefore had presumably a higher HRQoL than their counterparts that were dropping out of follow-up.

Although our sample size was larger (self-assessment form) or at the limit (parent proxy form) of the calculated minimum number, the low response rate may have affected the clinical strength of our results. Therefore, extrapolation of our results to a national perspective ought to be made with caution. A Brazilian multi-center study with a larger cohort is necessary to achieve a stronger statistical and especially clinical significance.

Two studies showed results similar to ours. One study conducted in the Russian Federation, using the same questionnaire as in the present study, showed statistically significant lower scores for PKU children when compared to healthy peers in all PedsQL core scales (34). Lower HRQoL scores, assessed by the Child Health Questionnaire (CHQ), were also demonstrated in a group of children and adolescents in Italy (35).

An increasingly low adherence to treatment over time as PKU patients reached school age and particularly adolescence was found in this study. This deterioration in treatment adherence is expected in PKU patients as well as in other chronic conditions that require diet therapy for many reasons. Adolescents and adults perceive dietary therapy as a constraint to their activities of daily living, find it hard to prepare protein substitutes, are more prone to social and peer group pressures, and may perceive their condition as stable (14,31,36).

This non-adherence could have influenced negatively HRQoL by reducing the intellectual capacity of PKU children and adolescents. We could not demonstrate, on the other hand, a correlation between adherence to treatment and quality of life in these patients. The lower scores obtained for almost all HRQoL scales by PKU children and adolescents in comparison to a normative population could not be attributed to non-adherence in the present study. This absence of correlation between adherence to treatment and HRQoL in PKU was also found by Cotugno et al. (35) in Italy. Cazzorla et al. (37), also from Italy, reached opposing results, finding an association between Phe levels and HRQoL scores in PKU. However, they included patients on BH4 treatment, and consequently their results are not comparable to our and Cotugno et al. (35) studies.

There is a scarcity of studies addressing the correlation between adherence to treatment and HRQoL in PKU children and adolescents. These two Italian studies are the few that directly concentrated on the subject.

Even though the correlation between compliance and HRQoL in PKU is debatable, there is solid evidence that PKU diet is hard to comply with (14,38), and, as a result, patients and their families consider it as a limitation to their daily life activities. This may have obscured any potential difference attributable to adherence, and may possibly have negatively influenced all HRQoL dimensions independently of achieving recommended phenylalanine levels.

Nevertheless, the importance of an adequate adherence to treatment at an early age on cognitive development and school performance cannot be overemphasized. The lower performance of Brazilian PKU children in executive functions (39), and intellectual capacity (40) are most probably due to the highly unsatisfactory adherence to diet treatment found in the reference centers around the country. The significant inverse relationship between parent proxy-report school functioning score and median Phe levels in the first year of life found in the present study (Figure 2) is a strong evidence of the impact of non-adherence in cognitive performance.

Lower scores were obtained for almost all HRQoL scales for self-reports and parent-proxy reports of PKU patients when compared to a normative sample of healthy Brazilian school children. Poor adherence to dietary treatment, ascertained by phenylalanine blood levels, was also found not to be optimal in the first year of life and tended to worsen, as patients grew older. Nonetheless, no significant differences in HRQoL scores were found between adherent and non-adherent children and adolescents. Failure of the healthcare system to ensure a regular supply of PKU metabolic formula to patients and the financial burden of acquisition of low protein food by families can be compounded by the well-known barriers to dietary compliance in PKU children and adolescents from Brazil affecting negatively all HRQoL dimensions irrespective of maintaining a satisfactory phenylalanine blood level. Few articles have addressed the impact of adherence to dietary treatment on HRQoL of patients with PKU and consequently further studies are urgently needed to clarify if adherence to diet therapy leads in fact to a better HRQoL, especially among children and adolescents.

## Supplementary material

Click here to view [pdf].
